# Maresins

**DOI:** 10.3390/biom16010139

**Published:** 2026-01-13

**Authors:** Fernanda Berrocal-Navarrete, Paz Marín-Sanhueza, Ramón Norambuena-González, Matías Quiñones San Martín, Francisca Herrera-Vielma, Daniel R. González, Jessica Zúñiga-Hernández

**Affiliations:** 1 Laboratory of Pharmacology and Physiology, Faculty of Health Sciences, University of Talca, Av. Lircay s/n, Talca 3460000, Chile; fberrocal18@alumnos.utalca.cl (F.B.-N.); pmarin19@alumnos.utalca.cl (P.M.-S.); ramon.norambuena@utalca.cl (R.N.-G.); mquinones@utalca.cl (M.Q.S.M.); francisca.ingbiotec@gmail.com (F.H.-V.); dagonzalez@utalca.cl (D.R.G.); 2 Doctorate in Biomedical Sciences Program, Faculty of Health Sciences, University of Talca, Av. Lircay s/n, Talca 3460000, Chile; 3 Institute of Biological Sciences, School of Biochemistry, University of Talca, Av. Lircay s/n, Talca 3460000, Chile; 4 Doctorate in Science Program, Mention in Research and Development of Bioactive Products, Institute of Chemistry of Natural Resources, University of Talca, Av. Lircay s/n, Talca 3460000, Chile

**Keywords:** omega-3, nutrition, pro-resolution, PUFAs

## Abstract

Polyunsaturated fatty acids (PUFAs), particularly omega-3 derivatives such as docosahexaenoic acid (DHA), are precursors of specialized pro-resolving mediators (SPMs) that actively orchestrate the resolution of inflammation. Among these, maresins (MaRs) have gathered increasing attention due to their potent immunomodulatory and tissue-regenerative properties. This review provides a comprehensive synthesis of the current knowledge on the biosynthesis, structural diversity, and biological functions of MaRs, with a focus on MaR1. We discuss the enzymatic pathways involved in the generation of MaR1, MaR2, MaRs conjugates in tissue regeneration (MCTRs), and maresin-like lipid mediators (MaR-Ls), highlighting their roles in modulating inflammatory responses, promoting phagocytosis, and restoring tissue homeostasis. Preclinical evidence from in vitro and in vivo models demonstrates that MaRs exert protective effects in a wide range of pathological contexts, including neuroinflammation, liver injury, cardiovascular dysfunction, pulmonary diseases, and metabolic disorders. Although their therapeutic promise is well-supported, key gaps remain in the understanding of MaRs biosynthesis, receptor specificity, and translational applicability. This review emphasizes the importance of advancing mechanistic and clinical research to fully harness MaRs as part of next-generation therapeutics in inflammation-driven diseases.

## 1. Introduction

The health benefits of polyunsaturated fatty acid (PUFAs) consumption have been recognized since 1971, when Bang, Dyerberg and Nielsen published an article about the lower levels of plasma lipids found in Eskimo populations (from the northern west coast of Greenland) compared to other populations (Danes and Eskimos living in Denmark) with similar fat intake. The difference was that Eskimos consumed mostly fish, seals and whales, in which the fat content is rich in long-chain PUFAs, particularly C18–C22 fatty acids, most of them omega-3 fatty acids [1]. These seminal findings laid the foundation for the current understanding of the nutritional and therapeutic relevance of omega-3 fatty acids, and to a lesser extent omega-6, fatty acids, stimulating extensive research into the biological roles and health benefits of PUFAs consumption.

**Figure 1:**
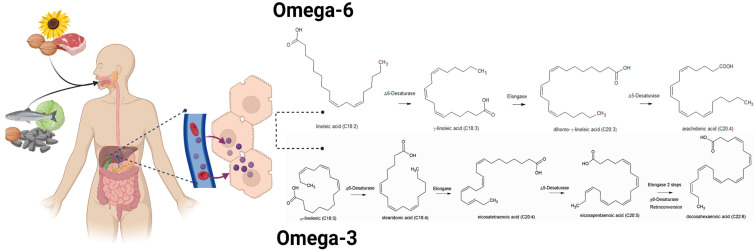
Biosynthetic pathways of major lipid mediators derived from dietary omega-6 (**top**) and omega-3 (**bottom**) fatty acids, highlighting the key enzymes involved in each conversion process.

Among PUFAs, which include omega-3 (n-3) and omega-6 (n-6) fatty acids, only linoleic acid (LA, C18:2n-6) and α-linolenic acid (ALA, C18:3n-3) are strictly essential, since mammals lack the Δ12 and Δ15 desaturases required for their synthesis [2]. From these, long-chain fatty acids precursors, such as arachidonic acid (AA; C20:4n-6), an omega-6 derivative, and eicosapentaenoic acid (EPA; C20:5n-3) and docosahexaenoic acid (DHA; C22:6n-3), both omega-3, can be synthesized through elongation and desaturation steps. However, this conversion is relatively slow, making dietary intake of LA and ALA necessary to maintain adequate levels needed for several processes [3], such as brain and retinal development [4,5], prevention of cardiovascular diseases and hepatoprotection [6,7,8]. Once incorporated into the organism, these fatty acids undergo elongation and desaturation reactions to form their respective derivatives such as AA for omega-6, and EPA and DHA for omega-3 (Figure 1) [2]. There is competition between omega-6 and omega-3 for the enzymes responsible for desaturation, in a way that an increase in omega-6 generates a decrease in the amount of omega-3 [9]. The modern diet is characterized by an unbalanced ratio of omega-6 to omega-3 fatty acids, especially LA, that can increase the production of proinflammatory eicosanoids such as prostaglandins, thromboxanes and leukotrienes derived from AA [9]. This metabolic imbalance contributes to a chronic low-grade inflammatory state, which is closely associated with metabolic disorders such as obesity and type 2 diabetes mellitus (T2DM) [10,11]. In recent years, the scientific focus has shifted from the preventive role of PUFAs toward understanding their active involvement in the resolution of inflammation through the generation of bioactive lipid mediators. Omega-3 PUFAs have been shown to actively promote the resolution of inflammation through the generation of specialized pro-resolving mediators (SPMs) derived from these fatty acids, including lipoxins (LXs), resolvins (Rvs), protectins (PDs), and maresins (MaRs) [12,13]. Among these, MaRs have emerged as promising bioactive molecules with significant therapeutic potential in pathological conditions such as asthma, hepatic injury, and cardiovascular diseases [14,15,16]. Therefore, this review focuses on the current research, biosynthetic pathways, and biological functions of MaRs, given their growing importance as novel therapeutic targets in inflammation (Figure 1).

## 2. Biosynthesis of Maresins

MaRs were discovered by Serhan et al. in 2009 [17]. These, along with other molecules of the same category such as PDs and Rvs, were described as SPMs, derived from EPA, and DHA [12]. The term “maresin” refers to *ma*crophage mediators in the *res*olution of *in*flammation (ma-res-in), reflecting its origin and biological function [18].

**Figure 2:**
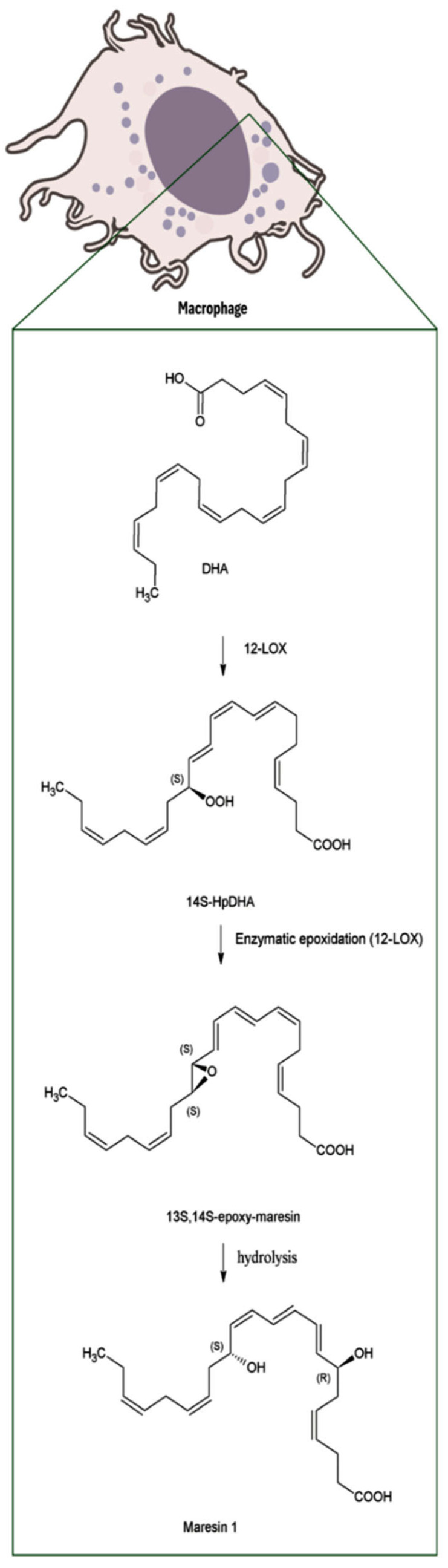
Biosynthesis of MaR1 from DHA in macrophages. Note: Abbreviations: Docosahexaenoic acid (DHA); 12-lipoxygenase (12-LOX); 14S-hydro (peroxy)-docosa-4Z,7Z,10Z,12E,16Z,19Z-hexaenoic acid (14S-HpDHA).

**Figure 3:**
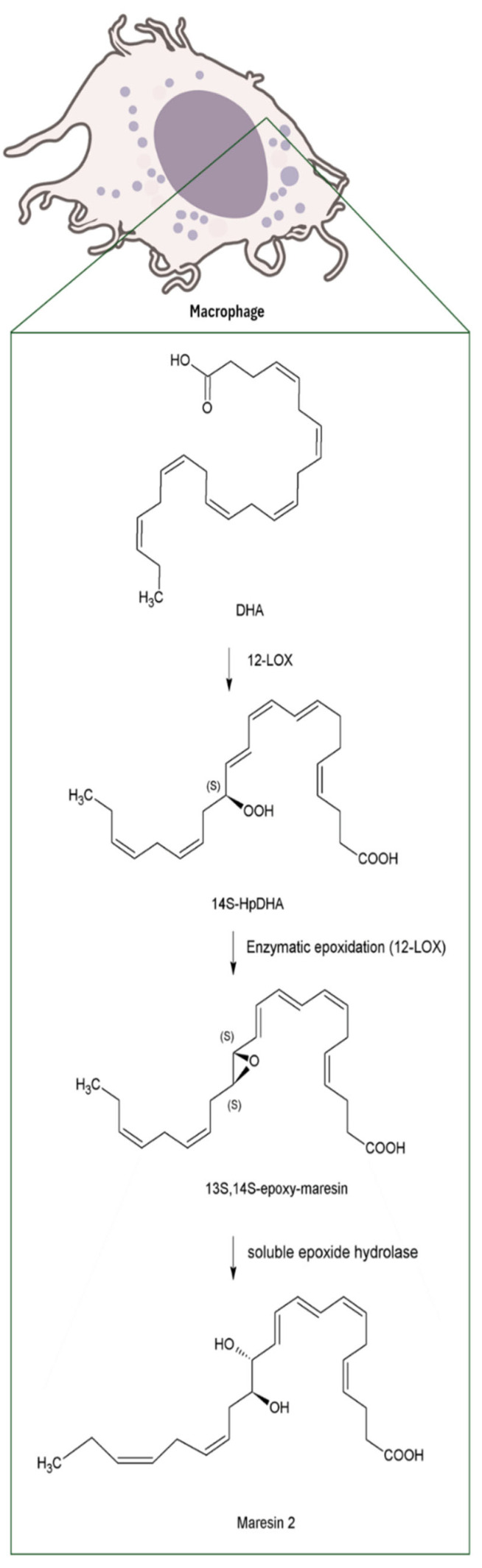
Biosynthesis of MaR2 from DHA in macrophages. Note: Abbreviations: Docosahexaenoic acid (DHA); 12-lipoxygenase (12-LOX); 14S-hydro(peroxy)-docosa-4Z,7Z,10Z,12E,16Z,19Z-hexaenoic acid (14S-HpDHA); soluble epoxide hydrolase (sEH).

**Figure 4:**
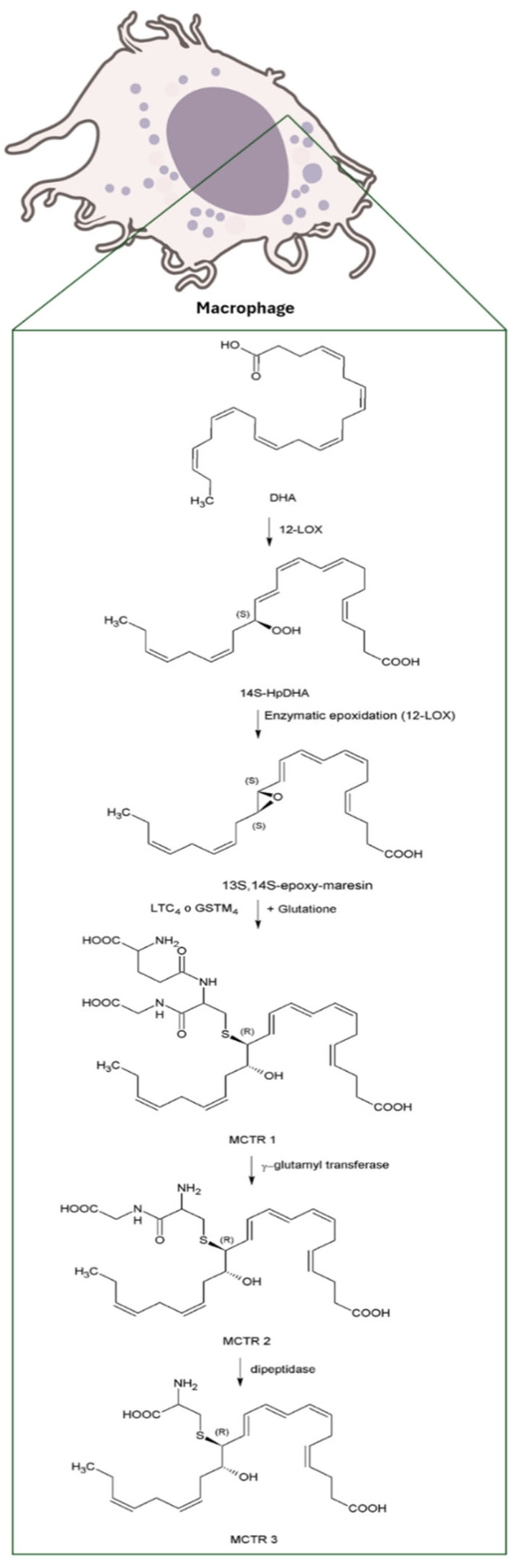
Biosynthesis of MCTR from DHA in macrophages. Note: Abbreviations: Docosahexaenoic acid (DHA); 12-lipoxygenase (12-LOX); 14S-hydro(peroxy)-docosa-4Z,7Z,10Z,12E,16Z,19Z-hexaenoic acid (14S-HpDHA); Leukotriene C4 synthase (LTC_4_ S); glutathione S-transferase MU4 (GSTM_4_).

**Figure 5:**
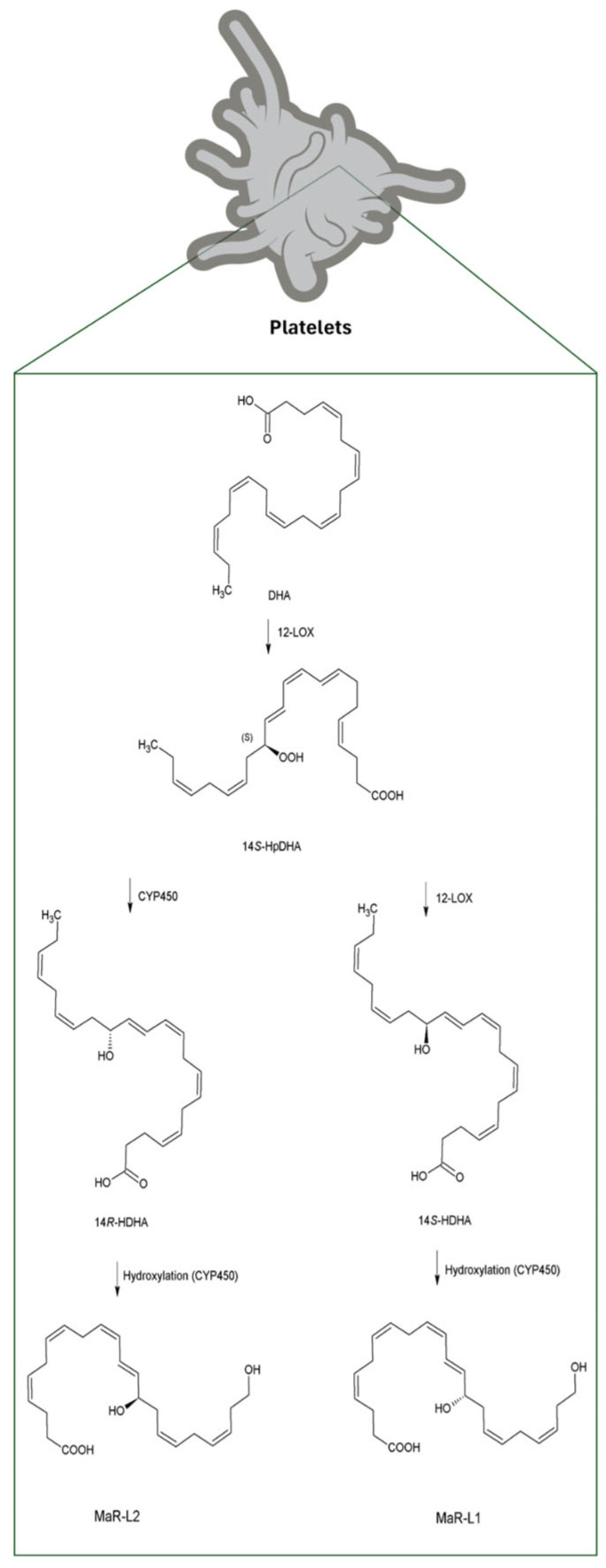
Biosynthesis of MaR-L from DHA in platelets. Note: Abbreviations: Docosahexaenoic acid (DHA); 12-lipoxygenase (12-LOX); 14S-hydro(peroxy)-docosa-4Z,7Z,10Z,12E,16Z,19Z-hexaenoic acid (14S-HpDHA); Cytochrome P450 (CYP450).

MaRs biosynthesis begins in macrophages with the 14-lipoxygenation of DHA by the human 12-lipoxygenase (12-LOX), that produces 14S-hydro(peroxy)-docosa-4Z,7Z,10Z,12E,16Z,19Z-hexaenoic acid (14S-HpDHA), a key intermediate in the MaRs pathway. Subsequently, the same enzyme promotes the formation of 13S,14S-epoxy-maresin (13,14-epoxide), an unstable intermediate that serves as precursor for MaRs metabolites [18,19]. Finally, through the sequential action of several enzymes, some of which remain to be fully characterized, the biosynthesis of the four MaRs subtypes described to date is completed. These different MaRs forms, along with their biosynthetic routes and biological activities, are discussed in detail in this review (Figure 2, Figure 3, Figure 4 and Figure 5).

## 3. Types of Maresins

To date, four main types of MaRs have been identified: MaR1; MaR2; MCTR and MaR-L [20]. They derive from the initial intake of omega-3 fatty acids and the subsequent lipoxygenation of DHA by the enzyme 12-LOX. Detailed information on their structural characteristics is provided in Table 1.

### 3.1. Maresin 1 (MaR1)

MaR1 is primarily synthesized by macrophages and exerts potent pro-resolving actions during inflammation. Its biosynthetic pathway begins with the formation of the intermediate 13,14-epoxy-maresin, which subsequently undergoes epoxide hydrolysis at the 13,14S-epoxy double bond. This reaction leads to the formation of a Z/E configuration, ultimately yielding 7R,14S-dihydroxydocosa-4Z,8E,10E,12Z,16Z,19Z-hexaenoic acid, commonly known as maresin 1 [18,21] (Figure 2).

### 3.2. Maresin 2 (MaR2)

MaR2, similarly to MaR1, limits infiltration of polymorphonuclear neutrophils and stimulates macrophages phagocytosis [22]. Its biosynthesis begins, with the 13S,14S-epoxy-maresin intermediate, then it is oxidized to 13R, 14S-dihydroxydocosahexaenoic acid (13R-14S-diHDHA), MaR2, by the action of soluble epoxide hydrolase (sEH), a key enzyme found in human macrophages [23] (Figure 3).

### 3.3. Maresin Conjugate in Tissue Regeneration (MCTR)

MCTRs are bioactive maresin-derived metabolites involved in tissue repair and cellular regeneration. The biosynthesis of these mediators occurs by lipoxygenation of DHA (see Figure 4), which produces 14-hydro(peroxy)-docosahexaenoic acid plus an intermediate epoxide that, through the catalysis of glutathione S-transferase MU4 (GSTM4) and leukotriene C4 synthase (LTC4 S) results in 13R-glutathione,14S-hydroxy-4Z,7Z,9E,11E,13R,14S,16Z,19Z-docosahexaenoic acid (MCTR1). Through the catalysis of γ-glutamyl transferase (GGT), 13R-cysteinylglycinyl,14S-hydroxy-4Z,7Z,9E,11E,13R,14S,16Z,19Z-docosahexaenoic acid (MCTR2) is generated [24,25,26] and, finally, by dipeptidase catalysis (DPEP), 13R-cysteinyl,14S-hydroxy-4Z,7Z,9E,11E,13R,14S,16Z,19Z-docosahexaenoic acid can be obtained [26].

### 3.4. Maresin-like Lipid Mediators (MaR-L)

Following the conversion of DHA into 14S-hydroperoxy-DHA (14S-HpDHA) by 12-lipoxygenase (12-LOX), the biosynthesis of MaRs and related lipid mediators can proceed through two distinct hydroxylation pathways. The 14S-hydroxylation pathway is initiated by 12-LOX, while an alternative 14R-hydroxylation is catalyzed by cytochrome P450 (CYP450) (Figure 5). Subsequently, 14S-hydroxydocosahexaenoic acid (14S-HDHA) undergoes a second hydroxylation by CYP450; this step has been attributed to CYP450 activity rather than 12-LOX, as suggested by recent studies indicating that multiple enzymatic systems may contribute to Mar-L biosynthesis, leading to the formation of 14,22-dihydroxydocosahexaenoic acid (14S,22-diHDHA), also known as MaR-L1. In parallel, MaR-L2 is synthesized through CYP450-mediated hydroxylation of 14R-HDHA, yielding 14R,22-diHDHA [27].

## 4. Conversion of DHA to Maresins: Evidence and Knowledge Gaps

Although, the biosynthesis of MaRs from DHA has been well described at the mechanistic level, quantitative data on the conversion rate remains unavailable. In addition to conversion efficiency, the synthesis of MaRs is influenced by several factors, including enzyme expression (12-LOX for MaRs, 15-LOX for Rvs), substrate availability (DHA vs. EPA), and cell type-specific signaling. Macrophages preferentially produce MaRs during resolution phases, whereas neutrophils may favor Rvs under acute inflammation. These decisions are modulated by receptor activation, intracellular redox status, and spatial enzyme organization within lipid rafts. Indeed, this process is tightly regulated and occurs primarily in macrophages, where the activity of 12-LOX plays a critical role in the conversion of DHA to MaRs. Importantly, recent evidence indicates that glutathione peroxidase (GPx) activity critically regulates MaRs synthesis. The formation of the 13S,14S-epoxy-maresin intermediate occurs only when GPx activity is insufficient to reduce the hydroperoxide precursor of its hydroxy derivative, favoring the epoxide pathway under conditions of partial GPx deficiency. This dependency explains why MaRs and PD1 are often difficult to quantify in tissues, as their levels are highly sensitive to cellular antioxidant status [28]. Several studies have employed liquid chromatography–tandem mass spectrometry (LC-MS/MS) to detect MaR1 both in vitro and in vivo models [23]. In neuronal cell cultures subjected to oxidative stress, MaR1 concentrations ranging from 1 to 100 nanomolar have demonstrated neuroprotective effects that surpass those of DHA, suggesting a functionally efficient conversion at this concentration range [29,30].

Although precise conversion percentages remain unavailable, the observed relationship between administered DHA concentrations (1–10 µM) and detected MaR1 levels (1–100 nM) [21] suggests a conversion efficiency in the range of approximately 0.01–1%, depending on the cellular and physiological context.

## 5. Preclinical Investigations

A growing body of preclinical research has explored the biological activity of MaRs in the context of human diseases. These studies, encompassing both in vitro and in vivo models, have been instrumental in elucidating the immunomodulatory, anti-inflammatory, and tissue-protective properties of MaRs. By simulating pathological conditions, these models provide valuable insights into the mechanisms through which MaRs exert their proresolving actions and support the development of novel therapeutic strategies.

### 5.1. In Vitro Studies

The functional characterization of MaRs has been extensively investigated using in vitro models, particularly those employing human-derived cells. These systems have proven essential for dissecting the cellular and molecular pathways modulated by MaRs, allowing researchers to evaluate their effects on immune cell behaviors, epithelial integrity, oxidative stress, and cytokine production. The following sections summarizes the current evidence on the biological actions of different MaRs subtypes in in vitro settings.

#### 5.1.1. Maresin 1

##### Central Nervous System

The central nervous system has emerged as a key target in the study of MaR1, particularly due to its relevance in neurodegenerative conditions such Alzheimer’s disease. Current evidence suggests that MaR1 exerts immunomodulatory effects on microglia, the resident immune cells of the brain that play a central role in the progression of neuroinflammation and neuronal damage. In this context, two studies have explored the impact of MaR1 on microglial responses to the amyloid-beta42 (AB_42_ peptide), a neurotoxic fragment derived from amyloid precursor protein that aggregates into plaques and triggers inflammatory responses in the brain. In the first of these, MaR1 (1 and 100 nM) was able to reduce proinflammatory markers such as CD33, activated CD11b, MHC-II, and CD86 and promoted phagocytosis of AB_42_ in CHME3 cells, a human microglial cell line [31]. This functional shift was further supported by studies in the human monocytic leukemia cell line (THP-1), and in human monocyte-derived microglia, where a micromolar dose of MaR1 increased the cellular uptake of AB_42_, reducing the secretion of inflammatory cytokines and chemokines, and reduced the CD40 marker and nuclear factor kappa-light-chain-enhancer of activated B cells (NF-κB) activity, reinforcing its role in the active resolution of microglial inflammation [32]. Beyond surface markers and cytokines profile, transcriptomic analyses in human monocyte-derived microglia showed that the treatment with MaR1 (5 µM) suppressed the expression of proinflammatory genes upregulated by AB_42_, including tumor necrosis factor alpha (TNF-α) and NF-κB, suggesting a pro-resolving effect associated with Alzheimer’s disease [33]. Interestingly, the neuroprotective potential of MaR1 extends beyond microglia. In a hybrid cell line created by fusion of mouse neuroblastoma cells with motoneurons-enriched embryonic spinal cord cells (NSC-34) subjected to oxidative stressors, MaR1 (10 nM) reduced reactive oxygen species (ROS) production and NF-κB activation. This suggested that MaR1 may also act directly on neuronal populations, contributing to cellular resilience under pathological conditions [30].

Taken together, these studies illustrate the multifaceted role of MaR1 in the central nervous system, where it modulates immune response, facilitates the clearance of neurotoxic aggregates, and protects neuronal integrity. These mechanism position MaR1 as a promising candidate for therapeutic strategies focused at mitigating neuroinflammation and neurodegeneration, particularly Alzheimer’s diseases.

##### Respiratory System

The respiratory system has emerged as another relevant target for MaR1, particularly in the context of acute and chronic inflammatory responses. In vitro models have provided compelling evidence of its ability to regulate epithelial and immune cells behavior under stress conditions. In human neutrophils exposed to lipopolysaccharide (LPS), pretreatment with MaR1 (1–100 nM) inhibited phosphorylation of survival signaling proteins, triggering proapoptotic signals and promoting the resolution of the inflammation [34]. This suggests that MaR1 may restore the physiological turnover of neutrophils, a process often impaired in chronic lung injury. In murine lung cells (MLE-12) incubated with LPS, MaR1 showed a concentration-dependent reduction in epithelial permeability, mostly effective at 100 nM. Importantly, MaR1 upregulated the expression of claudin-1 (CLDN1) and tight junction protein (ZO-1), both essential for the integrity of tight junctions, indicating a barrier-protective role that could be critical in preventing pulmonary edema and pathogens invasion [35]. Further insights were obtained from a model of airway inflammation induced by organic dust exposure. Using BEAS-2B bronchial epithelial cells pretreated with MaR1 (0–200 nM) for 24 h, Nordgren et al. showed that MaR1 reduced the activation of protein kinase C (PKC), and consequently decreased the production of TNF-α, IL-6 and IL-8 [36]. These findings reinforce the anti-inflammatory profile of MaR1 and its capacity to modulate epithelial signaling cascades. Beyond epithelial responses, MaR1 has also been evaluated in a tuberculosis model using monocyte-derived macrophages differentiated from human peripheral blood, stimulated with LPS or *M. tuberculosis*. Treatment with MaR1 (150 nM) resulted in a significant reduction in TNF-α production in both conditions. Additionally, MaR1 enhanced the antimicrobial activity of macrophages by lowering the bacterial load and inducing the production of bactericidal/permeability-increasing protein (BPI). Importantly, MaR1 promoted nuclear translocation of NF-κB p65 and nuclear factor erythroid 2-related factor 2 (Nrf2), suggesting a dual regulatory effect on inflammatory and antioxidant pathways [37].

In the case of the pulmonary system, the studies highlight the potential of MaR1 to modulate lung inflammation through different pathways, by restoring apoptosis, protecting epithelial integrity or decreasing the production of proinflammatory cytokines.

##### Oral Diseases

In addition to its action on major organ systems, MaR1 has demonstrated relevant effects in infectious and inflammatory disease of the oral cavity. Wang et al. investigated its action in macrophages derived from patients with localized aggressive periodontitis, showing a significant restoration of their phagocytic capacity of *P. gingivalis* and *A. actinomycetemcomitans*. Even at a concentration of 1 nM, phagocytosis increased by 37% and 65%, respectively, reaching levels comparable to healthy subjects and restoring homeostasis in chronic infections [38]. Complementarily, MaR1 has been studied in human bone marrow-derived mesenchymal stem cells (hBMMSCs), to explore new endodontic strategies for bacterial infections in pulpal and periapical pathology. In this model, hBMMSCs were stimulated with LPS and the treatment with MaR1 (1 to 100 nM) resulted in enhanced cell viability, proliferation, migration, survival, and reduced expression of inflammatory cytokines. The most potent effects were obtained when MaR1 was combined with RvE1 [39].

These findings highlight the dual role of MaR1 in oral pathologies where it promotes immune resolution and supports tissue regeneration through coordinated cellular mechanism.

##### Endocrine and Metabolic Diseases

MaR1 has demonstrated coordinated actions across multiple metabolic and regenerative contexts, including adipose tissue, bone, and bladder epithelium. In differentiated human adipocytes (hMSCs and hSPs), incubation with MaR1 (1–10 nM) promoted the expression of key adipokines such as adiponectin (ADIPOQ), dipeptidyl peptidase 4 (DPP-4) and cardiotrophin-1 (CT-1). In TNF-α induced inflammation, MaR1 (1–200 nM) partially restored the gene expression of adipokines, ADIPOQ, leptin (LEP), and DPP-4, suggesting a role in preserving adipocyte function in inflammatory diseases [40]. Likewise, MaR1 has been implicated in the modulation of ferroptosis in the context of diabetes-induced osteoblast dysfunction. In MC3T3-E1 osteoblastic cells, incubation with MaR1 (1–10 nM) partially restored the proliferative capacity and expression of osteogenic proteins, and enhanced the expression of NRF2, a key regulator of the antioxidant response linked to the inhibition of ferroptosis. These findings suggest that MaR1 may contribute to preserving osteoblast function in altered metabolic states by regulating pathways involved in oxidative stress and cell death [41]. In a bone repair model, MaR1 was evaluated in the crosstalk between macrophages and mesenchymal stem cell (MSCs), using a demineralized bone matrix scaffold model modified with MaR1 (at 150 nM) and 19S DNA aptamer (Apt19S). In this model, MaR1 activated PPARγ in macrophages, promoting their polarization toward the M2 phenotype and reducing proinflammatory mediators, which in turn enhanced MSC proliferation, migration, and osteogenic differentiation. The effects were abolished by PPARγ inhibition, confirming the dependence of this pathway [42]. Finally, in the bladder, MaR1 significantly accelerated wound closure in vitro and reduced inflammation in vivo within 3 days of cyclophosphamide (CP) induced injury [43].

Together, these findings illustrate the integrative role of MaR1 in modulating inflammation and promoting tissue repair across metabolic vulnerable systems. The experimental conditions and outcomes of the in vitro studies using MaR1 described above are summarized in Table 2, highlighting its consistent anti-inflammatory and regenerative effects across different cell types.

#### 5.1.2. Maresin 2

The benefits of MaR2 in in vitro models have only been reported in two studies to date, both related to their role in epithelial repair. In the first, the authors tested different doses of MaR2 in an intestinal wound healing model with HT29/B6 cells, where they showed no effects on the wound closure rate. However, when pre-incubated with proinflammatory cytokines, such as TNF-α and IFN-γ, MaR2 (50–200 nM) was able to promote an increase in wound closure at 24 h’ post-injury [44]. In the second, the effect of MaR2 was evaluated on rat-derived conjunctival goblet cells, where it was able to promote an increase in intracellular calcium concentration ([Ca^2+^]_i_), and stimulated the secretion of high molecular weight glycoconjugates, including mucin. These responses were mediated through G protein-coupled receptors (GPCRs) that involved the activation of PKC [22].

These studies suggest that MaR2 acts as a pro-reparative mediator that enhances epithelial and secretory function under inflammatory conditions, by modulating the secretion of proinflammatory cytokines and stimulating the production of glycoconjugates. Although limited, the current evidence on MaR2 in in vitro settings is summarized in Table 3, which highlights its role in epithelial repair and secretory function.

#### 5.1.3. Maresin Conjugate in Tissue Regeneration (MCTR)

In the case of MCTRs, there are also only two studies describing their benefits to date. In one of them, using primary macrophages incubated with MCTR1 (1 pM–10 nM) concentration-dependently increased phagocytosis of *Escherichia coli* (*E. coli*), promoting efferocytosis by neutrophils, a key mechanism for tissue regeneration. In the case of MCTR3, it produced a greater increase in phagocytosis of *E. coli* compared to MCTR2 and MCTR1, highlighting its potent anti-inflammatory and pro-resolving actions [26]. On the other hand, Chiang et al. investigated how these MCTRs could counteract the vascular response stimulated by leukotriene D4 (LTD_4_), using CHO cells transfected with CysLT1, an LTD_4_ receptor. In this case incubation with MCTR (10–100 nM) reduced the signaling induced by LTD_4_ [24].

Both approaches reinforce the potential of MCTRs as efficient mediators of tissue repair, by enhancing resolution mechanisms such as efferocytosis and phagocytosis or by inhibiting proinflammatory signaling pathways. The distinct actions of MCTR1-3 in promoting phagocytosis and modulating inflammatory signaling are illustrated in Table 4, which presents the main findings in vitro.

#### 5.1.4. Maresin-like Mediators (MaR-L)

Regarding MaR-L lipid mediators, to date, there are only two in vitro studies examining MaR-L effects. In one, the authors used macrophages derived from diabetic mice, characterized by a delay in the resolution of inflammation. Treatment with MaR-L (10 or 50 nM) promoted the restoration of the reparative activity of macrophages, facilitating cell migration, modulating the production of growth factors, and suppressing chronic inflammation, even at concentrations as low as 10 nM [27]. The second study by Thamizhchelvan et al. evaluated the effect of MaR-Ls (MaR-L1, MaR-L2 and MaR-L3) in the context of complex bacterial infections associated with traumatic burns, in combination with a bactericide on biofilms formed by strains of *Staphylococcus aureus*, *Pseudomonas aeruginosa* and *Escherichia coli*. The biofilms were treated with MaR-Ls (1–100 nM), alone or in combination with carbenicillin for 24 h. While treatment with MaR-Ls alone did not show significant effects, co-administration with the bactericide resulted in a significant reduction in bacterial cell viability [45].

These studies suggest that MaR-L is not only involved in the resolution of inflammation but may also be used as co-adjuvant of existing therapies in conditions where tissue repair or infection control is compromised. The emerging evidence on MaR-L mediators is presented in Table 5, showcasing their potential in restoring macrophage function and enhancing antimicrobial responses.

### 5.2. Studies in Animal Models of Human Diseases

In this context, in vivo studies have been essential to evaluate the therapeutic potential of MaRs. These models allow for the assessment of systemic responses to inflammation and tissue injury, providing insight into how MaRs modulate disease progression and promote resolution in physiologically relevant settings.

#### 5.2.1. Maresin 1

##### Liver

MaR1 has shown consistent hepatoprotective effects across a wide range of liver injury conditions, including acute inflammation, chronic fibrosis, and metabolic dysfunction. In carbon tetrachloride (CCL4)-induced acute liver injury, MaR1 administration 10–100 ng/mouse intraperitoneal (*i.p.*), reduced serum alanine aminotransferase (ALT) and aspartate aminotransferase (AST) levels, suppressed oxidative stress and lipid peroxidation, and decreased hepatic necrosis and apoptosis. These effects were associated with reduced expression of TNF-α, interleukin-6 (IL-6), interleukin-1 beta (IL-1β), monocyte chemoattractant protein-1 (MCP-1), cyclooxygenase-2 (COX-2), and inducible nitric oxide synthase (iNOS), and were mediated through inhibition of NF-κB and mitogen-activated protein kinase (MAPK) signaling pathways [46]. The relevance of this finding lies in the ability MaR1 to act early in the inflammatory cascade, potentially preventing the transition from acute damage to chronic liver dysfunction. In another acute model using lipopolysaccharide/D-galactosamine (LPS/D-GalN), MaR1 (50–100 ng/mouse, *i.p.*) not only attenuated systemic inflammation and reduced ROS, but also promoted macrophage polarization towards the M2 phenotype, and suppressed pyroptosis by decreasing the N-terminal fragment of gasdermin D (GSDMD-N), NLR family domain containing 3 (NLRP3), and IL-1β expression [47]. These results were complemented by in vitro experiments in cells stimulated with LPS where limited apoptosis was observed, and the expression of NLRP3, GSDMD-N and IL-1β was suppressed [47]. In another model of acute injury, hepatic ischemia/reperfusion (I/R), MaR1 treatment (5–20 ng/mouse, *i.p.*) significantly decreased several parameters associated with liver damage, including serum levels of ALT and AST, necrotic areas, and hepatocyte apoptosis in dose-dependent manner. Systemic inflammation was also reduced, as evidence by lower expression of IL-6 and IL-1B, suggesting that MaR1 could be exerting this effect at least in part through the ALX receptor (ALXR)/protein kinase B (Akt) signaling pathway [15].

In fibrotic liver models (chronic liver disease), such a as those induced by diethylnitrosamine (DEN), MaR1 normalized transaminase levels, improved hepatic architecture, and reduced inflammation and oxidative stress by inhibiting NF-κB translocation and activating Nrf2, TNF-α and IL-1β [48], suggesting that MaR1 may not only halts fibrotic progression but also promote tissue remodeling and recovery, a critical need in chronic liver disease management. In the context of obesity and no-alcoholic fatty liver disease, MaR1 ameliorated hepatic steatosis in both genetically obese (ob/ob) and diet induced obese (DIO) mice. MaR1 (2–10 μg/kg/day, *i.p.*, for 20 days) reduced hepatic triglyceride (TG) content, downregulated fatty acid synthase (FAS) and stearoyl-CoA desaturase-1 (SCD1), and increased phosphorylation of acetyl-CoA carboxylase (ACC), LC3-II expression and autophagic vacuole formation. In DIO mice, MaR1 (2 μg/kg/day, *i.p.*, or 50 μg/kg/day, oral gavage, for 10 days) produced similar effects, including reduced serum transaminases, and upregulated carnitine palmitoyltransferase 1A (CPT1A), acyl-CoA oxidase 1 (ACOX1), and autophagy-related genes Atg5 and Atg7. These effects were dependent on AMP-activated protein kinase (AMPK) activation, as AMPK inhibition abolished MaR1-induced gene expression changes [49]. In high-fat diet (HFD)-fed mice, MaR1 also suppressed endoplasmic reticulum (ER) stress stimulating AMPK and increasing sarco/endoplasmic reticulum Ca^2+^-ATPase 2b (SERCA2b) expression, thereby reducing hepatic lipid synthesis and steatosis [50]. Additionally, MaR1 protected hepatocytes from palmitate-induced lipotoxicity and hypoxia-induced ER stress by activating unfolded protein response (UPR) prosurvival mechanism and modulating specific microRNA signatures involved in protein folding and apoptosis [51].

Together, these findings establish MaR1 as a multifunctional hepatoprotective mediator capable of modulating inflammation, oxidative stress, pyroptosis, ER stress, lipid metabolism, and autophagy, with strong therapeutic potential for both acute and chronic liver diseases.

##### Cardiovascular Diseases

Heart

MaR1 has shown cardioprotective effects across diverse models of cardiac injury, acting through antioxidants, anti-inflammatory, and anti-ferroptotic mechanisms. In neonatal cardiomyocytes, MaR1 promoted physiological hypertrophy, by activating the retinoic acid-related orphan receptor alpha (RORα), which in turn induced the production of insulin-like growth factor 1 (IGF-1), and the triggered the phosphatidylinositol 3-kinase (PI3k/Akt) pathway [16]. This mechanism suggest that MaR1 may contribute to adaptative cardiac growth rather than pathological remodeling, offering a potential strategy to support cardiac development or recovery from injury. Similarly, in a murine model of myocardial infarction (MI), the treatment with MaR1 (10 ng/g for 28 days post-MI) reduced the cardiac remodeling and the incidence of arrythmias. These effects were associated with the activation of the Nrf2 pathway and attenuation of NF-κB [52], indicating that MaR1 may protect the myocardium by enhancing antioxidant defenses while suppressing proinflammatory transcriptional programs. Importantly, the reduction in arrhythmias suggests a stabilizing effect on cardiac electrophysiology, which could be clinically relevant in infarcted patients. In a model of I/R injury, MaR1 treatment (1 ng/mouse) decreased infarcted size and cardiomyocytes cell death by suppressing both apoptosis and pryroptosis, again associated with an inhibition of the NF-κB pathway and a reduction in the Silent Information Regulator Factor 2-related enzyme 1 (SIRT1) activity, thus inhibiting the activation of the inflammasome NLPR3 [53]. In the case of sepsis-induced cardiac dysfunction in mice, another model of acute injury, the administration of the MaR1 (100 ng/mouse) mitigated the cardiac impairment caused by LPS administration. This protective effect was attributed to reduced oxidative stress via activation of NRF2-HO pathway, and decreased inflammation, likely through modulation of cardiac macrophage activity [54]. More recently, MaR1 (10 ng/g) was evaluated in a model of cardiotoxicity generated by the chemotherapeutic doxorubicin, where it attenuated lipid peroxidation via the NRF2/GPX4 axis, reducing cardiomyocytes death by ferroptosis [55]. This is particularly relevant given the clinical limitations of doxorubicin due to its cardiotoxic profile.

Taken together, these findings highlight the ability of MaR1 exert positive effects multiple cardiac pathophysiological processes, offering protection through convergent molecular pathways that regulate oxidative damage, cell death, and inflammatory signaling.

##### Vasculature

Beyond its effects in other organs and systems, MaR1 has also been investigated in the vascular system, where it has shown a broad capacity to regulate vascular homeostasis. The first report in this context was in a model of neointimal hyperplasia by carotid ligation. Here, MaR1 (100 ng), similarly to Resolvin D2 (RvD2) (100 ng), reduced the proliferation of vascular smooth muscle cells (VSMCS), reduced neutrophils, and monocytes infiltration into the vessel wall. Notably MaR1 also promoted macrophage polarization toward the M2 phenotype [56], suggesting a shift toward a proresolving immune environment. Similarly, in a mouse model of atherosclerosis (*Apoe*^−/−^ mice fed a high-fat diet), co-administration of MaR1 and RvD2 (100 ng of each SPM), prevented the progression of the atherosclerotic lesion, reduced necrotic core size, and improved plaque stability. Interestingly, while MaR1 reduced macrophage content within the plaque, it simultaneously induced VSMCs proliferation, a paradoxical but beneficial effect in this context, as VSMCs contribute to fibrous cap formation and plaque stabilization [57].

Further evidence from cardiovascular models supports the vascular protective role of MaR1. In a mouse model of systemic arterial hypertension induced by angiotensin II (AngII) infusion, administration of MaR1 (2 μg/kg for 28 days) reduced blood pressure and vascular remodeling by acting on VSMCs through activation of the LGR6 receptor. MaR1 treatment inhibited VSMC proliferation, migration, and pyroptosis, leading to the reversal of pathological vessel wall changes [58]. Similarly, in a model of aortic aneurysm, MaR1 (4 ng/g body weight), also acting via LGR6, inhibited VSMC activation and attenuated aneurysm development. Interestingly, MaR1 also enhanced efferocytosis of dying VSMCs by macrophages, contributing to inflammation resolution and tissue repair [59].

Finally, in mouse models of pulmonary hypertension, MaR1 treatment showed therapeutic potential by ameliorating disease progression. The administration of MaR1 (1 μg per mouse, followed by 100 ng every two days, for three weeks) decreased pulmonary arterial pressure and right ventricular hypertrophy. It also decreased wall thickening and luminal narrowing, evidencing an improvement in this pathology, and inhibited pulmonary vascular smooth muscle cells proliferation by inhibiting STAT, Akt, ERK, and FoxO1 phosphorylation via the activation of the LGR6 receptor [60]. Also, it was reported that MaR1 ameliorated pulmonary hypertension in mice, acting on the ALX receptor, by reducing pulmonary smooth muscle cells proliferation [61].

##### Kidney Diseases

The kidney is another important organ affected by prevalent pathologies. Several studies MaR1 has shown protective effects in models of acute, ischemic, and metabolic kidney injury, acting through coordinated regulation of inflammatory, oxidative, and cell death pathways. First, in sepsis-induced kidney injury, the administration of MaR1 (5 μg/kg) decreased the expression of proinflammatory factors such as TNF-α, IL-1β, Il-6 and MCP-1 while it decreased the production of NOX4-derived ROS and the expression of IκBα and p65, key components of the NF-κB pathway [62]. MaR1 also induced renal protection via inhibition of the inflammasome, reducing endoplasmic reticulum stress, and hence pyroptotic cell death in a sepsis model of cecal ligation and puncture (0.04, 0.4 and 4 µg/kg) [63]. In a similar study, MaR1 (at 0.5 and 1 ng/mouse) acted via inhibition of the NF-κB/STAT3/MAPK pathways, resulting in decreased inflammatory cytokines production and increased survival rate [64]. In I/R injury, MaR1 (1.0 ng/mouse) targeted the TLR4, inhibiting the activation of NF-κB, attenuating inflammation and inducing the activation of Nrf2, improving the antioxidant defense [65]. Regarding diabetes, the potential of MaR1 to prevent the development of nephropathy was evaluated in mice treated with streptozotocin and a high fat diet (a type 2 diabetes model, DM2), at 4 µg/kg, for 14 weeks. Here, MaR1 reduced glomerular damage by decreasing the inflammation and the induction of the antioxidant response by overexpression of superoxide dismutase [66]. These findings are especially relevant given the limited therapeutic options for diabetic kidney disease and the central role of oxidative stress and inflammation in its progression.

##### Lung Diseases

The impact of Mar1 has been evaluated also in different models of pulmonary disease, where it exerts protective effects by modulating oxidative stress, inflammatory signaling and immune cells behavior. In lung I/R injury, MaR1 (1 ng/mouse) ameliorated pulmonary damage, inflammation and inflammatory cell infiltration associated with diminished ROS production, by enhancement of the antioxidant response of the Nrf-2 -heme oxygenase 1 (Nrf2-HO-1) pathway [67], suggesting that MaR1 activates endogenous antioxidant defenses, which are critical in limiting reperfusion-induced oxidative injury. MaR1 has also been shown to be effective in murine models of sepsis-associated lung injury. This was first described in a model of LPS-induced acute lung injury in mice. MaR1 (1 ng per mouse) reduced the damage by diminishing the inflammatory response, i.e., the surge of inflammatory cytokine and chemokines and neutrophils infiltration [68]. These observations were further reinforced in the same model by the analysis of the effects of MaR1 on the alveolar permeability, where it induced the expression of claudin-1 and ZO-1, thus reducing the pulmonary edema [35]. In addition, using the cecal ligation and puncture method of sepsis-associated acute lung injury, it was described that the treatment with MaR1 (at 0.5 and 1 ng/mouse) attenuated the inflammatory response by activation of the JAK2/STA3 and MAPK/NF-κB pathways [69]. Using the same model, but at 10 ng/mouse, MaR1 treatment reduced the pulmonary damage and induced the switch of alveolar macrophages to a M2 anti-inflammatory phenotype, through activation of peroxisome proliferator-activated receptor γ (PPAR-γ) [70]. Also, in this cecal ligation model, MaR1 (at 0.5 and 1 ng/mouse) reduced the levels of Th17 lymphocytes that favor the inflammatory response, while increasing the activation of Treg cells through the activation of the retinoid-related orphan nuclear receptor γ (ROR-γ) via the STAT3 pathway activation [71]. Finally, in this same model, at a dose of 1 ng/mouse, using single cell-sequencing, it was reported that MaR1 acts on a subpopulation of CXCL3 positive neutrophils, inhibiting the expression of the inflammatory pathway genes, reducing infiltration [72]. In addition, in a mouse model of asthma induced by ovalbumin, MaR1 (0.1, 1 and 10 ng/mouse) reduced inflammation dose-dependently by diminishing inflammatory cell recruitment, especially eosinophils and neutrophiles, associated with a reduction in NF-κB activation [14]. Finally, MaR1 was evaluated in a model of respiratory syncytial virus-induced lung inflammation. Here, MaR1 (10 ng/mouse) inhibited the viral aberrant transformation of Tregs cells and promoted interferon-β production, by activation LGR6 receptors. This blocked the production of IL-13, which is key to the transformation of Tregs lymphocytes into a prophlogistic phenotype [73].

##### Others

Beyond its effects on major organs, MaR1 has shown promising results in models of neuroinflammation, pancreatitis, and osteoarticular pain. In sepsis-associated encephalopathy (SAE) model, induced by cecal ligation and puncture, pretreatment with MaR1 (4n/g) attenuated the adverse effects of neuroinflammation by activating the SLC7A11/GPx4 ferroptosis pathway [74]. In acute (AP) and chronic (CP) pancreatitis induced by *i.p.* administration of caerulein (50 μg/kg), MaR1 treatment (2 ng) was evaluated. Here, MaR1 administration improved histopathological alterations, decreased the number of receptor-interacting protein kinase 3 (RIP3) and p-MLKL cells involved in necrosome activation, reduced macrophage infiltration and attenuated pancreatic fibrosis [75]. More recently the effects of MaR1 on osteoarthritis (OA)-like pain induced by injection with monosodium iodoacetate in animals was investigated. Eight weeks after injury, this treatment attenuated the pain-associated behavior, evidenced by increased paw withdrawal threshold and increased weight bearing. In addition, a reduction in CGRP expression, macrophage activation, and inflammatory cytokine levels were monitored. These findings suggest that MaR1 could directly modulate the functional response of neurons through the RORα mediated signaling pathway [76].

In short, MaR1 has been a focus of research in addressing various diseases affecting multiple organs. These studies position this molecule as a future therapeutic alternative for these conditions where only palliative options are available. To provide a comprehensive view of MaR1 systemic effects, Table 6 compiles the in vivo studies across various organs and disease models, emphasizing its therapeutic versatility.

#### 5.2.2. Maresin 2

MaR2 has shown a wide range of therapeutic effects in preclinical models of pain, inflammation, epithelial injury, and metabolic dysfunction. In murine models of *Bothrops jararaca* venom-induced pain and inflammation, MaR2 administered intraperitoneally at doses of 0.3–3 ng reduced mechanical and thermal hyperalgesia, restored hindpaw weight distribution, and decreased the levels of TNF-A, IL-1B, IL-6, myeloperoxidase activity, and superoxide anion production, while enhancing total antioxidant capacity and reducing hemorrhage and leukocyte infiltration [77]. In a model of trigeminal neuropathic pain, MaR2 (1–10 ng, intrathecally) inhibited neuronal activation in the trigeminal ganglion, reduced CGRP^+^ and c-Fos^+^ neurons, and normalized phospho NF-κB, supporting neuromodulator and anti-nociceptive potential in chronic pain conditions involving central sensitization [78]. In allergic airway inflammation and asthma, MaR2 reduced the presence of inflammatory cells such as IL-4, proinflammatory cytokines such as IL-1B and IL-18. Additionally, MaR1 treatment reduced oxidative stress markers such as malondialdehyde (MDA), while increasing levels of antioxidant enzymes superoxide dismutase (SOD) and glutathione synthetase (GHS), indicating a restoration of redox balance in lung tissue [79]. In models of colonic injury, MaR2 (2 ng/g, *i.p.*) accelerated mucosal wound healing following DSS-induced colitis and biopsy-induced epithelial damage. This treatment promoted epithelial migration via activation of focal adhesion kinase (FAK), Scr, paxillin, vinculin, and talin signaling [44]. In diet-induced obese mice, cold exposure and β3-adrenergic stimulation activated brown adipose tissue (BAT) to produce MaR2, which targeted hepatic macrophages, suppressed TNF-α, IL-1β, and NLRP3 inflammasome activation, and improved insulin sensitivity, linking MaR2 to metabolic inflammation resolution [80]. Finally, in zebrafish models exposed to *Loxosceles intermedia* venom, although MaR2 was not directly tested, the study highlighted systemic inflammation, oxidative stress, and behavioral alterations, reinforcing the relevance of pro-resolving mediators like MaR2 in venom-induced pathology [81]. In conjunctival goblet cells, MaR2 (0, 1–10 nM) increased intracellular calcium and stimulated mucin secretion via GPCR- mediated pathways, suggesting a role in ocular surface homeostasis and dry eye diseases [22].

Together, these findings establish MaR2 as a multifunctional lipid mediator capable of resolving inflammation, modulating pain, promoting epithelial repair, restoring redox balance, and improving metabolic outcomes, with strong translational potential across diverse pathophysiological conditions. The anti-inflammatory and antioxidant properties of MaR2 in respiratory disease models are summarized in Table 7, highlighting its potential in allergic airway inflammation.

#### 5.2.3. Maresin Conjugate in Tissue Regeneration (MCTR)

Another biomolecule part of the MaRs family is MCTR, where the strongest evidence focusses on the use of MCTR1. In a model of acute lung injury, MCTR1 administration (10–100 ng/mouse) led to a reduction in morphological parameters of damage, including decreased inflammatory cell infiltrate, interstitial edema, among others (ref). These effects were accompanied by lower levels of proinflammatory cytokines TNF-α and IL-1β. They also determined that the action of this pro-resolving mediator is mediated by the ALX receptor activation, a G-protein-coupled receptor that induces increase in intracellular cAMP [82]. In the kidney, MCTR1 was evaluated in a model of septic acute kidney injury, where it attenuated ferroptosis by upregulating glutathione peroxidase 4 (GPX4) levels and downregulating prostaglandin-endoperoxide synthase 2 (PTGS2).

These molecular changes correlated with improved renal morphological changes and restoration of kidney function [83]. In a model of sepsis-induced cardiac injury, MCTR1 treatment improved cardiac function by decreasing the left ventricular end-systolic volume, increased after LPS administration. In addition to improving left ventricular fractional shortening and ejection fraction, MCTR1 treatment also decreased the mRNA expression of proinflammatory factors such as IL-1β, IL-6 and TNF-α [84]. In LPS-induced lung injury, MCTR1 treatment inhibited reverse transendothelial migration of neutrophils, reducing systemic inflammation and pulmonary damage [85]. It also protected the pulmonary endothelial glycocalyx by upregulating syndecan-1 and heparan sulfate, thereby preserving vascular integrity and reducing permeability [86]. In bleomycin-induced pulmonary fibrosis, MCTR1 administered from day 7 to 21 post-injury reversed epithelial-to-mesenchymal transition (EMT), reduced collagen deposition, improved lung function, and increased survival rates [87]. In remifentanil-induced hyperalgesia, MCTR1 treatment (10–100 ng, *i.p.*) alleviated pain by regulating mitochondrial fission though suppression of dynamin-related protein 1 (DRP1) expression and restoring mitochondrial morphology in dorsal root ganglia neurons [88]. MCTR3, acting via the ALX/PINK1 signaling pathway, reduced mitochondrial dysfunction and oxidative stress in LPS-induced lung injury, improving histological outcomes and reduced inflammatory cytokines [89]. In influenza A virus–primed mice challenged with *Streptococcus pneumoniae*, administration of MCTR1–3 (10 ng, *i.p*.) restored alveolar macrophage migration, reduced CXCL1 secretion, and gene expression, thereby reducing bacterial load and lung inflammation [90]. Mechanistically, cysteinyl-MCTRs activated TRAF3 via cAMP–PKA signaling, increasing IL-10 production, and enhancing macrophage phagocytic capacity, with effects confirmed in both planarian and mammalian models [91]. Additionally, continual efferocytosis was shown to be metabolically primed by MCTR production in macrophages. Apoptotic cell breakdown upregulated 12-lipoxygenase, leading to MCTR synthesis, which enhanced Ras-related C3 botulinum toxin substrate 1 (Rac1)-mediated glycolysis and glucose uptake. Genetic ablation of MCTR synthesis impaired efferocytosis, confirming autocrine and paracrine roles for MCTR in metabolic reprogramming, sustained clearance and repair [92].

Together, these findings establish MCTR1–3 as multifunctional lipid mediators that orchestrate resolution of inflammation, mitochondrial protection, immune reprogramming, and tissue regeneration across diverse models of sterile and infectious injury. The organ-specific protective effects of MCTR in acute inflammatory conditions are detailed in Table 8, which includes studies in lungs, kidney, and heart models.

#### 5.2.4. Maresin-like Lipid Mediators (MaR-L)

The last group of molecules in the MaRs family are MaR-L, which have recently gained attention for their roles in immune modulation and tissue repair (see Table 9). To date, only a few studies have evaluated their biological effects in vivo. In a transgenic 5xFAD mouse model of Alzheimer’s disease, Shrivastava et al. investigated the long-term intranasal administration of MaR-L1 at 100 ng/mouse, three times per week from 1.5 to 9 months of age. MaR-L1 treatment significantly reduced amyloid-β plaque burden in the hippocampus and cortex, preserved cholinergic neurons (NauN^+^ and ChAT^+^), and decreased cleaved caspase-3 expression associated with apoptosis. It also suppressed M1 microglial markers (CD68^+^), promoted M2 polarization (Iba1^+^/Arg-1^+^), and reduced neutrophil infiltration while enhancing claudin-5 expression, suggesting improved blood–brain barrier integrity and neuroimmune homeostasis [93]. Complementarily, Hong et al. reported that MaR-L lipid mediators are endogenously produced by leukocytes and platelets and can restore the reparative function of macrophages impaired by diabetes. In this study, macrophages derived from diabetic mice exhibited reduced efferocytosis and tissue repair capacity, which was reversed by treatment with MaR-Ls (10–100 nM). These mediators enhanced Rac1-dependent cytoskeletal remodeling and glucose uptake, reprogramming macrophages toward a pro-resolving phenotype. Lipidomic analysis confirmed the presence of MaR-L1, MaR-L2, and MaR-L3 in human leukocytes and platelets, and their biosynthesis was shown to be dependent on 12-lipoxygenase activity [27].

Together, these findings position MaR-Ls as emerging lipid mediators capable of restoring immune resolution, promoting neuronal survival, and enhancing macrophage reparative functions, with promising implications for neurodegenerative and metabolic diseases.

**Table 9:** Experimental design and outcomes of the in vivo study using MaR-L1 in a mouse model of Alzheimer’s disease and diabetes (Db/Db). The table highlights its impact on neuronal survival, microglial phenotype, blood–brain barrier integrity and metabolic parameters.

Year	Organ	Pathogeny	Model	Impact	Reference
2024	Brain	Alzheimer’s	Male mouse 5xFAD C57BL/6J	↑ Amyloid beta. MaR-L preserved the neuronal population. ↑ Cells ChAT^+^, ↓ Cells caspase-3^+^, ↓ Iba-1 ↓ Microglial cells CD68^+^. MaR-L restored the microglial phenotype. ↑ Partially branched microgrill cells, ↑ Microgrill cells type M2, ↓ Neutrophil infiltrate, ↑ Claudin-5. MaR-L improves the integrity of the blood–brain barrier	[93]
2014	Adipose tissue	Type 2 diabetes-induced macrophage dysfunction	Diabetic mice (Db/Db)	↑ Efferocytosis, ↑ Tissue repair capacity, ↑ Glucose uptake, ↑ Rac1-dependent cytoskeletal remodeling, ↑ Pro-resolving macrophage phenotype, ↑ Expression of M2 markers, ↓ Pro-inflammatory cytokines TNF-α, IL-6.	[27]

### 5.3. Studies in Humans

Although there are currently no clinical trials evaluating the direct administration of MaRs in humans, several studies have investigated their endogenous presence in various tissues and diseases. Among these, plasma levels of MaR1 have been the most extensively studied, particularly in metabolic disorders, where resolution mechanisms are often impaired. One of the earliest studies by Skarke et al. (2015) [94] assessed the formation of bioactive lipids following fish oil supplementation at high (21 g/day) and standard (4 g/day) doses. While an increase in the erythrocyte membrane ratio of EPA and DHA relative to AA was observed, SPMs such as MaR1 remained below detection limits, even after inflammation was induced via LPS administration, suggesting that the mere availability of precursors may not be sufficient to trigger SPM biosynthesis in vivo, especially under controlled inflammatory conditions. Similarly, in subjects with metabolic syndrome, omega-3 fatty acids intake did not alter plasma MaR1 levels, and precursor molecules such as 18-hydroxyeicosapentaenoic acid (18-HEPE), 17-hydroxydocosahexaenoic acid (17-HDHA), and 14-HDHA were also reduced [94]. This reduction in precursors bioavailability suggests a compromised biosynthetic capacity for SPMs in metabolic syndrome, potentially limiting the endogenous production of MaR1 despite omega-3 supplementation. It also raises questions about enzymatic activity and substrate accessibility in metabolically altered tissues. In contrast, a later study in obese individuals supplemented with marine oil enriched in 14-HDHA, 17-HDHA, and 18-HEPE (2 g/day for one month), showed a 4.7-fold increase in plasma MaR1 levels [95], possibly due to persistent inflammation in the study population. In another clinical study involving T2DM and diabetic foot ulcer (DFU) patients, plasma MaR1 levels were significantly lower compared to normoglycemic individuals, with negative correlations to body mass index (BMI), systolic blood pressure (SBP), low-density lipoprotein cholesterol (LDL-C), fasting plasma glucose (FPG), glycated hemoglobin (HbA1c), and homeostasis model assessment for insulin resistance (HOMA-IR) [96]. In acute respiratory distress syndrome (ARDS), lower MaR1 levels were associated with prolonged mechanical ventilation and intensive care unit (ICU) stay, while higher levels correlated with shorter ICU stays (<7 days), suggesting that defective resolution of inflammation may contribute to ARDS pathophysiology [97]. In NAFLD, serum MaR1 levels were significantly reduced compared to healthy controls, with positive correlations to albumin (ALB), high-density lipoprotein cholesterol (HDL-C), and negative associations with age, BMI, triglycerides, and hepatic transaminases (ALT, AST) [98]. In atherosclerotic cardiovascular disease (ASCVD), higher MaR1 levels were associated with reduced ASCVD risk, potentially mediated by LDL-C [99]. In women with coronary microvascular dysfunction (CMD), MaR1 levels were significantly lower compared to reference groups, while precursors such as EPA, DHA, and 18-HEPE were elevated, suggesting impaired enzymatic conversion of SPMs [100]. Omega-3 supplementation during pregnancy increased precursors levels at birth, but this effect was not sustained at 12 years of age, and MaR1 was undetectable despite elevated 14-HDHA. In preeclampsia (PE), although no significant differences in MaR1 levels were found between pregnant and non-pregnant women, the ratio of leukotriene B4 (LTB4) to MaR1 was markedly lower in PE patients, indicating an imbalance in inflammatory resolution [101]. Interestingly, a group of researchers investigated the effect of moderate alcohol consumption on plasma concentrations of SPMs in patients with T2DM. The study reported elevated SPM levels in T2DM subjects, suggesting a possible homeostatic response. However, no significant changes were observed in MaR1 or other SPMs following alcohol intake [102].

Although most human evidence has focused on MaR1, other members of the MaRs family, such as MCTRs, have also been detected endogenously in humans. One study examined allergic airway inflammation using human lung tissue and found that healthy tissue exhibited higher levels of MCTR1, MCTR2, and MCTR3 compared to diseased tissue. Upon incubation with MCTR1, metabolic conversion to MCTR2 and MCTR3 was observed, indicating active interconversion. MCTRs significantly blocked the action of leukotriene D4 (LTD4), revealing potential pro-resolving mechanisms in pulmonary responses that may be disrupted by disease [103]. A second study conducted in patients with rheumatoid arthritis identified all three MCTR family members in plasma. Each showed negative correlations with markers of systemic and joint disease activity, with statistically significant associations for MCTR3. These findings support the hypothesis that MCTRs may play a protective role in the resolution of chronic inflammation [104].

These studies expand the scope of MaRs research in humans, highlighting not only MaR1 but also MCTR1–3 as endogenous mediators potentially involved in the resolution of chronic inflammation and immune regulation across metabolic, respiratory, and autoimmune conditions.

## 6. Conclusions and Projections

MaRs, a family of SPMs derived from DHA, have emerged as potent regulators of inflammation, tissue repair, and resolution of pathological processes. Among them, MaR1 stands out due to its broad therapeutic spectrum, exhibiting anti-inflammatory, pro-resolving, and cytoprotective effects across multiple organs, including the liver, heart, lungs, kidneys, and central nervous system. Preclinical studies consistently show the ability of MaR1 to modulate key signaling pathways such as NF-κB, STAT3, and PI3K/Akt, while promoting macrophage polarization, enhancing phagocytosis, and preserving epithelial and endothelial barrier integrity.

Beyond MaR1, other members of the MaRs family including MaR2, MCTR1–3, and MaR-L1/2, have shown promising bioactivities, particularly in epithelial repair, immune modulation and resolution of chronic inflammation. However, their mechanisms of action remain less well-characterized. Emerging evidence suggests potential involvement of receptors such as LGR6, ALX, and GPCRs, as well as intracellular pathways like PPAR-γ, Nrf2, and RORγ, but further studies are needed to clarify their biosynthetic regulation, receptor specificity, and pharmacokinetics.

A particularly compelling aspect of MaRs is their ability to modulate specific immune cell types, including CXCL3^+^ neutrophils, Th17/Treg lymphocytes, and epithelial cells in several tissues. This cellular specificity suggests that MaRs may offer a more targeted and physiological approach to resolving inflammation compared to conventional anti-inflammatory drugs. Moreover, their influence on processes such as ferroptosis, pyroptosis, and efferocytosis opens new therapeutic avenues in degenerative, autoimmune, and metabolic diseases.

Despite the strong preclinical foundation, several challenges remain for clinical translation. These include dose optimization, molecular stability, delivery strategies, and the identification of biomarkers of resolution. The development of synthetic analogs, controlled-release formulations, and receptor-specific agonists may help overcome these barriers and accelerate the integration of MaRs into therapeutic protocols.

From a clinical perspective, MaRs offer promising strategies for managing chronic inflammatory diseases and acute conditions. Current approaches under investigation include synthetic analogs, receptor-specific agonist, and targeted delivery systems to enhance stability and bioavailability, aiming to harness their pro-resolving properties for therapeutic benefit.

Taken together, MaRs represent a novel class of bioactive lipids with the potential to reshape the treatment landscape for inflammatory and degenerative diseases. Their ability to resolve, rather than merely suppress, inflammation positions them as key candidates in the design of next-generation therapies that are both effective and aligned with the body’s natural healing processes.

## Figures and Tables

**Table 1:** Types of MaRs and their specifications.

Common Name	Chemical Formula	IUPAC Designation	PubChem Number
MaR1	C_22_H_32_O_4_	(7R,14S)-dihydroxy-(4Z,8E,10E,12Z,16Z,19Z)-docosahexaenoic acid	60201795
MaR2	C_22_H_32_O_4_	(13R,14S)-dihydroxy-(4Z,7Z,9E,11E,16Z,19Z)-docosahexaenoic acid	101894912
MCTR1	C_32_H_47_N_3_O_9_S	(13R)-S-glutathionyl-(14s)-hydroxy-(4Z,7Z,9E,11E,16Z,19Z)-docosahexaenoic acid	122368871
MCTR2	C_27_H_40_N_2_O_6_S	(13R)-S-cysteinylglycinyl-(14S)-hydroxy-(4Z,7Z,9E,11E,16Z,19Z)-docosahexaenoic acid	122368872
MCTR3	C_25_H_37_NO_5_S	(13R)-S-cysteinyl-(14S)-hydroxy-(4Z,7Z,9E,11E,16Z,19Z)-docosahexaenoic acid	122368873
MaR-L1	C_22_H_31_O_4_^−^	(4Z,7Z,10Z,12E,14S,16Z,19Z)-14,22-dihydroxydocosahexaenoate	126456468
MaR-L2	C_22_H_31_O_4_^−^	(4Z,7Z,10Z,12E,14R,16Z,19Z)-14,22-dihydroxydocosahexaenoate	126456470

Note: Abbreviations: Maresin 1 (MaR1); Maresin 2 (MaR2); Maresin-like lipid mediators (MaR-L) and Maresin conjugate in tissue regeneration (MCTR).

**Table 2:** Summary of in vitro studies evaluating the effects of MaR1 on various cell types. The table includes stimuli, doses, exposure times, and observed responses, emphasizing MaR1’s anti-inflammatory and proresolving actions.

Cell Line	Stimulus	[MaR1]	Time	Response	Reference
CHME3	1 µg/mL of Aβ_42_	0–100 nM	0–6 h	↑ phagocytosis of Amyloid β_42_.	[31]
Human monocyte-derived microglia and THP-1 cells	5 µM Aβ_42_	5 µM	24 h	↑ phagocytosis of Amyloid β_42_. ↓ CD40 and NF-κB.	[32]
Human monocyte-derived microglia	5 µM Aβ_42_	5 µM	4 h	Inflammatory effect ↓ TNF-α and NF-κB.	[33]
NSC-34 motor cells	Oxidative stress/SOD1 G93A, TDP-43 A315T mutation	1–1000 nM	-	↓ ROS reduction. ↓ NF-κB activation. Neuronal protection.	[30]
Primary human neutrophils	Lipopolysaccharides	1–100 nM	1–3 h	Restoration of phagocytosis.	[34]
MLE-12 cells	Lipopolysaccharides (1 µg/mL)	0.1–100 nM	30 min	↓ Epithelial permeability, ↑ Claudin-1, Zo-1.	[35]
BEAS-2B cells	Organic powder 5%	0–200 nM	-	↓ PKC, ↓ TNF-α, IL-6, IL-8.	[36]
hMSC and hSP cells	TNF-α	1–200 nM	24 h	Partial reverse lof disruption in ADIPOQ, LEP, DPP-4 and FNDC5.	[40]
MC3T3-E1 cells	High glucose plus palminate	1–10 nM	-	↑ Proliferation, ↑ Osteogenic proteins, ↑ NRF2; ↓ Dysfunction induced by the diabetic environment.	[41]
Peripheral blood macrophages	*P. gingivalis* and *A. actinomycetemcomitans*	0, 1–10 nM	-	↑ phagocytosis.	[38]
Monocyte-derived macrophages	LPS/*M. tuberculosis*	150 nM	1 h	↓ TNF-α ↑ BPI induced translocation of NF-κB p65 and Nrf2 to the nucleus	[37]
hBMMSC	LPS	100 nM	-	↑ TGF-β1, IL-10, IL-4 ↓ IFN-γ, RANKL y TNF-α	[39]
Bone repair model (demineralized bone matrix scaffold + MaR1 + Apt19S)	Macrophage-MSC interaction	150 nM	-	Activation PPARγ in macrophages, ↓ Proinflammatory mediators, ↑ MSC proliferation, migration and osteogenic differentiation	[41]
Bladder model (epithelial cells and in vivo CP injury)	Cyclophosphamide		3 days	↑ Accelerated wound closure, ↓ inflammation	[42]

**Table 3:** Summary of in vitro studies using MaR2, focusing on its effects on epithelial wound healing and mucin secretion under inflammatory conditions.

Cell Line	Stimulus	[MaR2]	Time	Response	Reference
HT29/B6 cells	Scratch injury	50–200 nM	24 h	↑ wound closure.	[44]
Globet cells	Without external stimulus	0.1 nM–10 nM	2 h	↑ [Ca^2+^]_i_ ↑ Secretion of glycoconjugates.	[22]

**Table 4:** Experimental parameters and out of in vitro studies using MCTR 1–3. The table highlights their role in enhancing phagocytosis, efferocytosis, and suppressing leukotriene-mediated responses.

Cell Line	Stimulus	Concentration	Time	Response	Reference
Primary leukocytes (macrophages and neutrophils)	*E. coli*	MCTR1, MCTR2, MCTR3 1 pM-10nM	-	↑ phagocytosis and efferocytosis.	[26]
CysLT1-transfected CHO cells	LTD_4_	MCTR 10–100 nM	-	↓ of LTD_4_-induced signals.	[24]

**Table 5:** Summary of in vitro studies evaluating MaR-Ls including their effects on macrophage repair activity and bacterial biofilm viability.

Cell line	Stimulus	Concentration	Time	Response	Reference
Macrophages	Scratch injury	MaR-L 10 o 50 nM	-	↑ repair activity of macrophages. ↓ in chronic inflammation.	[27]
(Not applicable)/Biofilms of clinical strains	Carbenicillin (±MaRLs)	MaRL1, MaRL2, MaRL3 1–100 nM	24 h	↓ in bacterial viability.	[45]

**Table 6:** Compilation of in vivo studies using MaR1 in models of liver, heart, kidney, lung, brain, and bone diseases. The table includes experimental models, pathologies, and observed protective effects.

Year	Organ	Pathogeny	Model	Impact	Reference
2016	Liver	Acute liver injury	BALB/c mice	↓ ALT/AST, oxidative stress, necrosis, apoptosis, TNF-α, IL-6, IL-1β, MCP-1, COX-2, iNOS, via NF-κB/MAPK inhibition.	[46]
2021	Liver	Acute liver injury	C57BL/6 mice	↓ ROS, Systemic inflammation, NLRP3, GSDMD, IL-1β, ↑ M2.	[47]
2021	Liver	Hepatic I/R injury	Male C57BL/BJ	↓ ALT/AST, necrosis/apoptosis, IL-6, IL-1β via ALXR/Akt pathway.	[15]
2021	Liver	Liver fibrosis	Male rats Sprague-Dawley	↓ Inflammation and oxidative stress, ↓ NF-κB, Nrf2.	[48]
2018	Liver	Obesity and NAFLD	Ob/Ob and Diet-Induced Obese Mice	↓ TGs, FAS/SCD1, ↑ ACC phosphorylation, LC3-II, autophagy, CPT1A, ACOX1.	[49]
2018	Liver	NAFLD	Male C57BL/6J mice	↓ ER stress, ↑ SERCA2b pathway AMPK activation.	[50]
2017	Liver	Lipotoxicity	Male C57BL/6J mice	↑ UPR survival pathway, modulation of miRNAs related to protein folding and apoptosis	[51]
2021	Heart	Cardiac hypertrophy	Male mice C57BL/6J	↑ Cardiomyocyte cell surface, ↑ IGF-1 production that activates P1EK/AKT. Producing physiological hypertrophy. ↑ Overexpression of the enzyme arachidonate 12-lipoxygenase in post-myocardial infarction patients.	[16]
2022	Heart	Myocardial infarction	Male mice C57BL/6J	↓ Cardiac remodeling and arrhythmias, ↑NRF2, ↓NF-κB signaling.	[52]
2025	Heart	Cardiac I/R	Male mice C57BL/6J	↓ Heart failure size. ↑ Cardiac function. Structural recomposition of myocardial tissue. ↓ IL-1β y TNF-α. NF-κB, ↓ Pyroptosis. ↓ NLRP-3.	[53]
2021	Heart	Sepsis-induced cardiac dysfunction	Mice C57BL/6J	↓ Cardiac dysfunction, ↑ NRF2-HO pathway, ↓ Inflammation.	[54]
2025	Heart	Cardiotoxicity	Mice C57BL/6J	↓ Lipid peroxidation through NRF2/GPX4, ↓ Ferroptosis-mediated cardiomyocyte death.	[55]
2016	Vasculature	Neointimal hyperplasia	Mice	↓ VSMC proliferation, neutrophil/monocyte infiltration, ↑ M2 macrophages	[56]
2016	Vasculature	Atherosclerosis	Mice apoE ^−/−^	↓ Lesion progression, plaque vulnerability, VSMC proliferation, ↑ M2 macrophages.	[57]
2020	Vasculature	Hypertension	Mice C57BL/6J	↓ Blood pressure, vascular remodeling, pyroptosis via LGR6 receptor. ↓ Pyroptosis.	[58]
2022	Vasculature	Aortic aneurysm	Mice C57BL/6J	↓ VSMC activation and aneurysm development ↑ efferocytosis by macrophages	[59]
2022	Vasculature	Hypertension	Adult male mice C57BL/6J	↓ Pulmonary pressure, RV hypertrophy, wall thickening via LGR6- mediated inhibition of STAT AKT, ERK, FOxO1.	[60]
2023	Vasculature	Hypertension	Mice and Rats	↓ VSMC proliferation pathway ALX activation.	[61]
2021	Kidney	Acute kidney injury associated with sepsis (LRA-S)	Male C57BL/6J mice	↓ Inflammation, IL-6, IL-1B, TNF-A, and MCP-1, ↓ BAX and caspase-3, ↑ ATP production, ↓ free oxygen radicals (ROS), ↓ Phosphorylation of IkBa and p65.	[62]
2024	Kidney	Sepsis	Male BALB/C mice	↓ Inflammasome, ER stress, pyroptosis.	[63]
2019	Kidney	Sepsis	Male C57BL/6J mice	↓ of the NF-κB/STAT3/MAPK pathways, ↓ Inflammatory cytokines, ↑ Survival.	[64]
2019	Kidney	Ischemia/reperfusion	Male C57BL/6J mice	↓ NF-κB, ↑ Nrf2 activation, antioxidant defense, ↓ Inflammation.	[65]
2022	Kidney	Diabetic nephropathy	Male C57BL/6J mice	↓ Glomerular damage, inflammation, ↑ SOD, antioxidant response	[66]
2017	Lung	I/R lung injury	BALB/c mice	↓ Pulmonary inflammation, ROS via NRf2 HO-1 pathway.	[67]
2014	Lung	Acute lung injury	BALB/c mice	↓ Cytokines and chemokines, neutrophil infiltration.	[68]
2016	Lung	Barrier function	Male BALB/c mice	↑ Claudin, ZO, ↓ Edema and permeability.	[35]
2020	Lung	Sepsis-associated acute lung injury	Male C57 mice	↓ Inflammation via JAK2/STAT3 and MAPK/NFκB	[69]
2020	Lung	Sepsis-associated acute lung injury	Male BALB/c mice	↑ M2 macrophages pathway ↑ PPARγ.	[70]
2020	Lung	Sepsis	Male C57BL/6 mice	↓ Th17, ↑ Treg via RORγ/STAT3 activation.	[71]
2022	Lung	Sepsis	Male C57BL/6	↓ CXCL3 neutrophil, ↓ Infiltration	[72]
2021	Lung	Asthma	Female BALB/c mice	↓ Eosinophil and neutrophil infiltration, ↓ NFκB.	[14]
2023	Lung	Inflammation	BALB/cByJ and FoxP3^eGFP^ mice	↓ Treg aberrant transformation, ↑ IFN-β pathway LGR6, ↓ IL-13.	[73]
2024	Brain	Sepsis-associated encephalopathy	Rat Sprague-Dawley	↓ Proinflammatory cytokines from MaR1 administration. MaR1 prevents spatial learning difficulties in mice with sepsis-associated encephalopathy.	[74]
2025	Bones	Osteoarthritis	Mice	↑ in endogenous MaR1 due to treadmill exercise. ↓ MMP13 synthesis.	[76]
2021	Pancreas	Acute and chronic pancreatitis (caerulein-induced)	ICR male mice	↓ RIP3/p-MLKL, macrophage infiltration and fibrosis	[75]

**Table 7:** Experimental conditions and therapeutic outcomes of in vivo studies using MaR2 in murine models of asthma and allergic airway inflammation.

Year	Organ	Pathogeny	Model	Impact	Reference
2025	Peripheral tissue	Pain/inflammation	Swiss Female mice	↓ Hyperalgesia ↓ TNF-α, IL-1β, IL-6, MPO activity, Hemorrhage, leukocyte infiltration ↑Total antioxidant capacity	[77]
2023	Nervous system	Neuropathic pain	Male Wistar rats and Swiss mice	↓ Neuronal activation, CGRP^+^, and c-FOs^+^ neurons Normalized pNF-κB	[78]
2022	Lung	Asthma	Female mice BALB/c	↓ Neutrophil infiltration. ↓ IL-4, IL-1β, IL-18, MDA ↑ SOD, GSH	[79]
2023	Colon	Epithelial injury/colitis		↑Mucosal repair ↑ FAK/Src/paxillin/vinculin/talin	[44]
2022	Liver	Obesity and metabolic inflammation	C57BL6/J mice	↓ TNF-α, IL-1β, NLRP3 Improved insulin sensitivity and glucose tolerance	[80]
2022	Whole organism	Inflammation	Zebrafish		[81]
2022	Eye	Epithelial dysfunction	Male albino Sprague-Dawley rats	↑ Intracellular Ca^2+^, mucin secretion ↑ Stimulated mucin secretion via GPCR mediated pathways	[22]

**Table 8:** Summary of in vivo studies using MCTR1 in models of acute lung, kidney, and cardiac injury. The table outlines molecular and functional outcomes, including inflammation, ferroptosis, and cardiac performance.

Year	Organ	Pathogeny	Model	Impact	Reference
2020	Lung	Acute lung injury	Male rats Sprague-Dawley	Improvement in histopathological changes in lung tissue, ↓ TNF-a, IL-1B and IL-10, ↑ Alveolar fluid clearance, Na+/K+-ATPase activity, cAMP concentration in lungs, P-Nedd4-2.	[82]
2021	Kidney	Acute septic kidney injury	Male mice C57BL/6	↑ GPX4, GSH and Nrf2, ↓ PTGS2, ↓ MDA ↑ mitochondrial length. ↑ Renal function, ↓ Serum creatinine, and blood urea nitrogen. Suppression of ferroptosis. Improvement of pathogenic changes.	[83]
2021	Heart	Sepsis-induced cardiac dysfunction	Male mice C57BL/6	↓ B natriuretic peptide. ↓ IL-6, IL-1B, IL-17A, TNF-a, CCL2, CCL7, CXCL1, and G-CSF. Promotes downregulation of CXCL1 and G-CSF. ↓ Neutrophil recruitment and infiltration.	[84]
2025	Lung	Lung injury	Male mice C57BL/6	Inhibited reverse transendothelial neutrophil migration, ↓ Systemic inflammation, pulmonary damage	[85]
2020	Lung	Acute lung injury	Mice	↓ TNF-α, IL-1β, IL-6, ↑ Syndecan 1, heparin sulfate, ↓ Permeability, Preserved vascular integrity.	[86]
2021	Lung	Pulmonary fibrosis	Male mice C57BL/6	↓ Collagen deposition, ↑ Survival, Reversed EMT.	[87]
2025	Nervous system	Hyperalgesia	Rats	↓ Pain, ↓ DRP1, NR2B, ↓ MDA and ROS Restored mitochondrial morphology.	[88]
2021	Lung	Lung injury	C57BL/6 mice	↓ Mitochondrial dysfunction, ↓ Oxidative stress, ↓ Cytokines.	[89]
2022	Lung	Secondary bacterial infection	Male mice C57BL/6	↓ CXCL1, ↓ Bacterial load and inflammation.	[90]
2023	Immune system	Inflammation resolution	Mac ^alox12/15 KO^ Mice	↑ IL-10, ↑ Macrophage phagocytosis.	[91]
	Macrophages	Efferocytosis and metabolic reprogramming		↑ 12-LOX, ↑ Rac-1, ↑ Glycolysis.	[92]

## Data Availability

No new data were created or analyzed in this review.

## Abbreviations

PUFAs	Polyunsaturated fatty acids
SPMs	Specialized pro-resolving mediators
MaRs	Maresins
MaR1	Maresin 1
MaR2	Maresin 2
MCTR	Maresin conjugates in tissue regeneration
MaR-L	Maresin-like lipid mediators
LA	Linoleic acid
ALA	α-linolenic acid
AA	Arachidonic acid
EPA	Eicosapentaenoic acid
DHA	Docosahexaenoic acid
LXs	Lipoxins
RVs	Resolvins
PDs	Protectins
12-LOX	12-Lipoxygenase
14S-HpDHA	14S-hydro(peroxy)-docosa-4Z,7Z,10Z,12E,16Z,19Z-hexaenoic acid
sEH	Soluble epoxide hydrolase
GSTM4	Glutathione S-transferase MU4
LTC4S	Leukotriene C4 synthase
GGT	γ-glutamyl transferase
DPEP	Dipeptidase catalysis
CYP450	Cytochrome P450
14S,22-diHDHA	14,22-dihydroxydocosahexaenoic acid
iNOS	Inducible nitric oxide synthase
COX2	Cyclooxygenase 2
AB_42_	Amyloid beta 42 peptide
MHC-II	Major histocompatibility complex class 2
CHME3	Human microglial cell line
THP-1	Human monocytic leukemia cell line
NSC-34	Motor neurons
LPS	Lipopolysaccharide
MLE-12	Epithelial Cells
CLDN1	Claudin 1
ZO-1	Protein zonula occludens-1
BEAS-2B	Immortalized human bronchial epithelial cell line
PKC	Protein kinase C
hMSCs	Human mesenchymal stromal/stem cells
hSPs	Heat shock Protein
Nrf2	Erythroid-derived nuclear factor 2
